# *n*-Butylidenephthalide Modulates Autophagy to Ameliorate Neuropathological Progress of Spinocerebellar Ataxia Type 3 through mTOR Pathway

**DOI:** 10.3390/ijms22126339

**Published:** 2021-06-13

**Authors:** Jui-Hao Lee, Si-Yin Lin, Jen-Wei Liu, Shinn-Zong Lin, Horng-Jyh Harn, Tzyy-Wen Chiou

**Affiliations:** 1 Everfront Biotech Inc., New Taipei City 22180, Taiwan; juihaolee@gmail.com (J.-H.L.); emily8302102102@gmail.com (S.-Y.L.); coldwee@gmail.com (J.-W.L.); 2 Department of Life Science, Graduate Institute of Biotechnology, National Dong-Hwa University, Hualien 97447, Taiwan; 3 Bioinnovation Center, Buddhist Tzu Chi Medical Foundation, Hualien 97002, Taiwan; Shinn-Zong@tzuchi.com.tw; 4 Department of Neurosurgery, Hualien Tzu Chi Hospital, Buddhist Tzu Chi Medical Foundation, Tzu Chi University, Hualien 97002, Taiwan; 5 Department of Pathology, Hualien Tzu Chi Hospital, Buddhist Tzu Chi Medical Foundation, Tzu Chi University, Hualien 97002, Taiwan

**Keywords:** MJD, SCA3, Purkinje cell, PolyQ, atxain-3, toxic fragment, autophagy, mTOR

## Abstract

Spinocerebellar ataxia type 3 (SCA3), a hereditary and lethal neurodegenerative disease, is attributed to the abnormal accumulation of undegradable polyglutamine (polyQ), which is encoded by mutated ataxin-3 gene (*ATXN3*). The toxic fragments processed from mutant *ATXN3* can induce neuronal death, leading to the muscular incoordination of the human body. Some treatment strategies of SCA3 are preferentially focused on depleting the abnormal aggregates, which led to the discovery of small molecule *n*-butylidenephthalide (*n*-BP). *n*-BP-promoted autophagy protected the loss of Purkinje cell in the cerebellum that regulates the network associated with motor functions. We report that the *n*-BP treatment may be effective in treating SCA3 disease. *n*-BP treatment led to the depletion of mutant *ATXN3* with the expanded polyQ chain and the toxic fragments resulting in increased metabolic activity and alleviated atrophy of SCA3 murine cerebellum. Furthermore, *n*-BP treated animal and HEK-293*^GFP-ATXN3-84Q^* cell models could consistently show the depletion of aggregates through mTOR inhibition. With its unique mechanism, the two autophagic inhibitors Bafilomycin A1 and wortmannin could halt the *n*-BP-induced elimination of aggregates. Collectively, *n*-BP shows promising results for the treatment of SCA3.

## 1. Introduction

Polyglutamine (polyQ) diseases are a group of genetic neurodegeneration caused by the excess of cytosine-adenine-guanine (CAG) repeats that encode a long polyQ tract in the translated proteins [1,2,3,4,5,6]. Misfolding and aggregation of the expanded polyQ proteins can selectively damage the neurons in distinct brain areas. There is increasing evidence suggesting the severe loss of Purkinje cells and irreversible cerebellar atrophy in most spinocerebellar ataxias (SCAs) [7,8,9]. Clinical symptoms of the SCAs including motor dysfunction/incoordination, gait ataxia, dysarthria, dysphagia, nystagmus, and cerebellar oculomotor disorder are rapidly progressive and ultimately fatal [2,10]. In addition, SCA type 3 (SCA3), also known as Machado-Joseph disease (MJD), is the most common autosomal dominantly inherited type of SCAs, and its mutation was mapped to chromosome 14q32.1 [2,11,12].

The accumulation of polyQ expansion (56–86 repeats) within the ataxin-3 gene (*ATXN3*) in the neurons was demonstrated to play an important role in the pathogenesis of SCA3 [12,13,14,15]. Calpain-mediated cleavage and translocation of *ATXN3* in the undegradable aggregates, e.g., polyQ expanded mutant *ATXN3* (m-*ATXN3*) and toxic fragments (TFs), into the nucleus in neurons can lead to degeneration of the Purkinje cells [16,17]. The excessive TF formation is highly associated with the decreased efficiency of the protein elimination [12,18,19]. In a normal neuron, *ATXN3* is involved in the editing of poly-ubiquitin chains to regulate protein degradation as a function of deubiquitinating enzyme [20,21] and is associated with cytoskeletal formation [22], dendritic differentiation [9], cell apoptosis, and protection against polyQ proteins in vivo [23]. These functions are attributed to the unique structure of the protein that is composed of Josephin domain with the capability of deubiquitination at the N terminus and the C terminal portion containing ubiquitin-interacting motifs (UIM) and polyQ regions [24,25]. Recent works revealed that *ATXN3* can interact with beclin-1 or LC3C/GABARAP through the polyQ region or the Josephin domain, respectively, as key players in autophagosome formation [26,27,28]. Beclin-1 regulated autophagosome formation can be particularly inhibited in the in vitro and the in vivo models of *ATXN3* depletion. Competitive polyQ tracts and polyQ region of the *ATXN3* with deubiquitinating activity for the beclin-1 can affect the autophagic process, and this may result in inefficiency and impairment in autophagic elimination of toxic aggregates.

Autophagy, the fusion of autophagosome and lysosome, is one of the pivotal protein degradation processes for removing unnecessary and/or abnormal proteins, preventing unusual accumulation and recycling crucial substances to sustain the physiological function [27,29]. In the autophagic process, the kinase mammalian target of rapamycin (mTOR) is a major regulator [30,31,32]. mTOR activity is modulated by the complicated interaction of the upstream regulators including AMP-activated protein kinase (AMPK), protein kinase B (AKT), and extracellular signal-regulated protein kinase 1/2 (ERK1/2). Sequentially, inhibition of mTOR triggers beclin-1 up-regulation and light chain protein 3-I (LC3-I) conversion into a lipidated active form, LC3-II. The interplay within the protein complex occurs at the isolated membranes and ends with the p62 interacting with LC3-II to complete the autophagosome formation. When autophagy is impaired or disrupted, many studies revealed that accumulation of aggregated proteins are observed in the various neurodegenerative diseases [27,33,34]. Highlighting the important role of autophagy and implying the drug targets in SCA3 disease, the therapeutic molecule may be accordingly developed.

Some compounds were isolated from the extracts of *Angelica sinensis*, a traditional Chinese herbal medicine, to evaluate cytotoxicity in cancers [35,36]. Many studies revealed the lead compound *n*-butylidenephthalide (*n*-BP) for its various functions in anti-tumor growth, anti-aggregation of platelets, and autophagic regulation in neurodegenerative disease [37,38,39,40]. Given the intriguing role of *n*-BP in autophagy and relieving neurodegenerative diseases, we evaluated the effect of *n*-BP on the *ATXN3* with 84 glutamine repeats transgenic mouse models by animal behavior analysis and imaging approaches. *n*-BP can ameliorate neuropathological progress after the disease onset and uncoordinated behavior and gait ataxia occurred. Treatment of SCA3 animals with *n*-BP protects their Purkinje cells from loss, reflected by increased metabolic activity and alleviated atrophy of the murine cerebellum. Notably, autophagy through the expression of beclin-1 and LC3B-II can efficiently eliminate abnormal aggregated proteins, although the mechanism of action of *n*-BP remains to be elucidated. We show that *n*-BP can be used for mitigating SCA3 disease through the mTOR inhibition to promote autophagy, thereby protecting the cerebellar Purkinje cells.

## 2. Results

### 2.1. n-BP Effectively Improved Motor Function in SCA3 Animals

**Figure 1:**
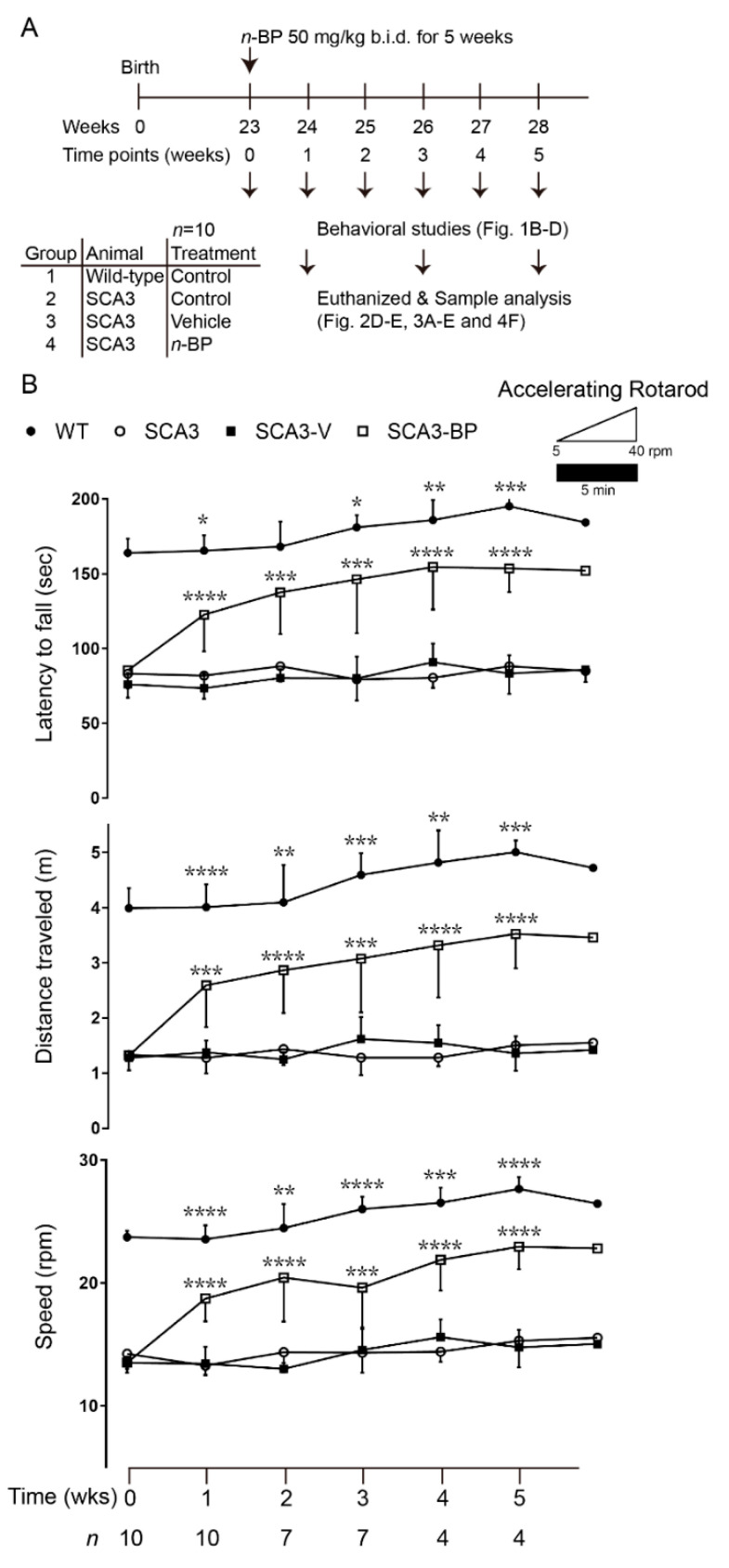

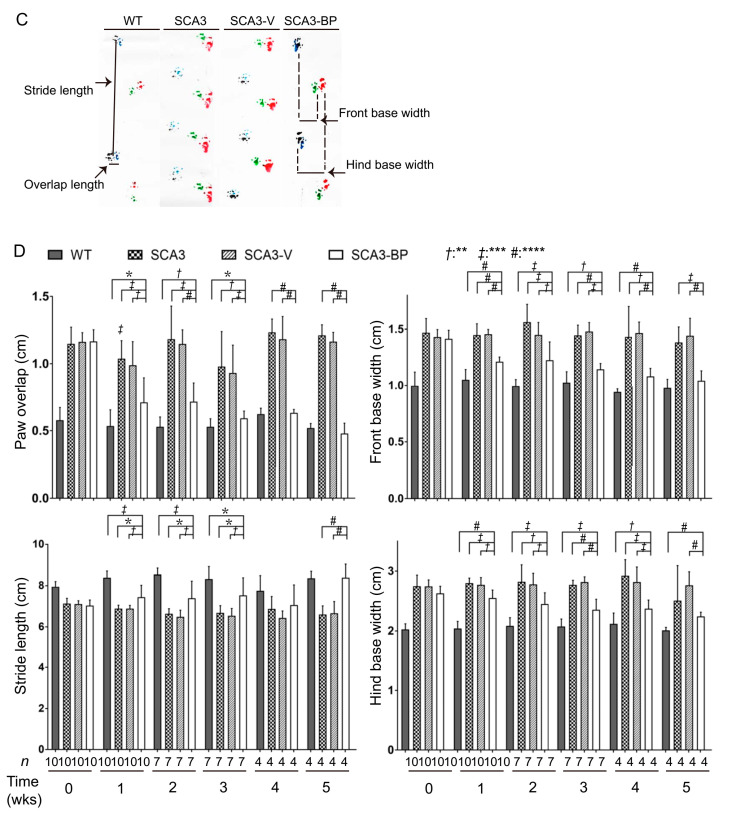
Administration of *n*-BP significantly alleviated impairments of motor coordination and ameliorated gait/locomotion in the SCA3 mouse model. (**A**) The representative designed study showing 23-week-old wild-type and SCA3 mice were divided into four groups (each *n* = 10 at the beginning): WT, SCA3, vehicle, or *n*-BP (50 mg/kg b.i.d.) treated with SCA3 (SCA3-V or SCA3-BP) for 5 weeks. Behavior studies were performed every week, and the studied animals were euthanized at first, third, and fifth weeks for sample collection and indicated analysis. (**B**) A significantly improved motor coordination of the *n*-BP treated SCA3 mice in accelerating (0–40 r.p.m. within 5 min) rotarod test was recorded. The *n*-BP treated SCA3 mice had better motor performance and balance compared to SCA3’s non-treated and vehicle groups. Asterisk presents the calculated *p* value of SCA3-BP versus WT or SCA3-V. To figure out the best response to *n*-BP during the treatment, small groups of 1–2 mice were additionally prepared for the behavioral evaluation at the sixth week. (**C**,**D**) Representative images of footprint patterns displaying the gait of *n*-BP administered SCA3 mice were similar to WT animals at the fifth week (28-week-old mice). Based on the 5 weeks’ footprinting test, SCA3-BP group presented improved coordination with shorter footprint overlap and longer stride length than vehicle or non-treated animals. Moreover, the long front and the hind base width in SCA3 and SCA3-V groups were presented. The 5 weeks of *n*-BP treatment made a significant improvement on balance of SCA3 mice (†: **, ‡: ***, #: ****). Animal number for the statistics was indicated as *n*. Data represent mean ± s.d.; * *p* < 0.05, ** *p* < 0.01, *** *p* < 0.001, **** *p* < 0.0001 (Student’s *t*-test).

The SCA3 mouse model investigated in this study displayed cerebellar degeneration and motor deficits at 13–14 weeks of age [18]. After that, the locomotion and the gaits of SCA3 mice were obviously deteriorated when evaluated via a rotarod test. *n*-BP oral administration was used in the 23-week-old mice with a progressive symptomatic status of the disease. To analyze the effects of *n*-BP on SCA3, murine behaviors were evaluated every week, and the animals were sacrificed to obtain whole brain samples to perform the molecular analysis that was based on the schedule (Figure 1A).

After five weeks of treatment (Figure 1B), SCA3 mice with *n*-BP exposure (SCA3-BP) presented a significantly improved performance in motor function and balance, determined by the accelerating rotarod tests. Conversely, the vehicle/non-treated SCA3 mice (SCA3-V or SCA3) quickly fell off the rotarod (top panel). Analyses of the traveled distance (middle panel) and the speed of rotarod (bottom panel) of SCA3 and SCA3-V mice falling off also presented an impressive improvement of the motor function in the SCA3-BP group. While evaluating motor performance at the sixth week among the SCA3 groups (*n* = 1–2), the best response to *n*-BP could be found at weeks four/five. To assess murine gaits in turn, analysis of footprint patterns was studied (Figure 1C and calculated in 1D). One to two weeks after the beginning of the *n*-BP treatment, the SCA3 mice exhibited a gradually improved footprint overlap (upper left panel of Figure 1D), and the behavior outcomes close to the wild-type (WT) mice group were found at the fourth to fifth week. The stride length was consistently longer in WT and SCA3-BP than vehicle or non-treated SCA3 mice (bottom left panel). In addition, incoordination and imbalance of the longer width of front and hind base were largely improved in the *n*-BP exposed SCA3 mice in comparison with SCA3 or SCA3-V groups from 1–5 weeks (top and bottom right panels). These evaluations of animal gaits suggest that *n*-BP significantly benefited the motor function. Taken together, 5 weeks of *n*-BP treatment alleviated the severe motor deficits in SCA3 mice.

### 2.2. n-BP Ameliorated Cerebellar Neuropathology of SCA3 Mice

**Figure 2:**
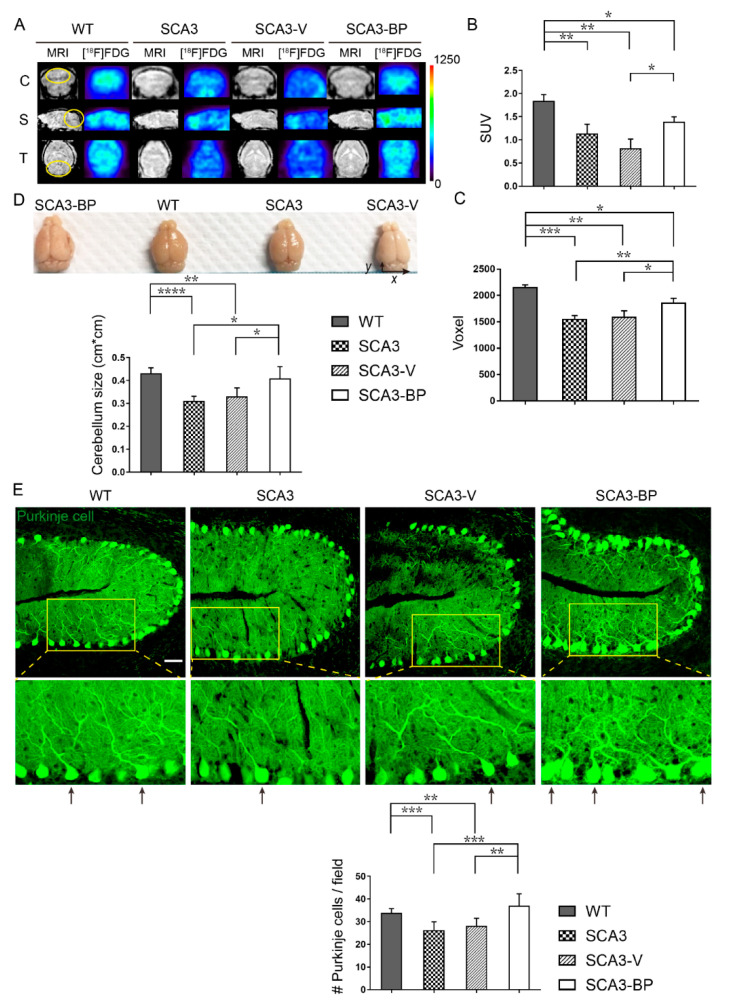
*n*-BP protected the Purkinje cells from loss to increase the metabolic activity in the cerebellum in the SCA3 mice. (**A**) The representative PET ([^18^F]FDG) and MR images, in coronal (**C**), sagittal (S), and transverse (T) views, of SCA3 mice showing that *n*-BP treatment for 12 weeks could alleviate the impairment of glucose analogue metabolism and restore the cerebellar function (yellow ROI). Each group contained three 35-week-old mice for the analysis. (**B**) The panel presents the values corresponding to the standard uptake value (SUV) based on the in vivo imaging of glucose metabolic activity (yellow ROI of Figure 2A). (**C**) Histogram indicated the MR evaluated cerebellar size in voxel, reflecting the cerebellar atrophy in SCA3 or SCA3-V animals. (**D**) The images exhibit the whole brain of WT and SCA3 mice with indicated treatments for five weeks. Statistical results suggested that the *n*-BP treatment could mitigate the atrophy of cerebellum, and no difference in cerebellum size (x × y = cm × cm) was found between WT and SCA3-BP groups (at 28 weeks of age). Three animals were used for the statistical data. (**E**) Cerebellar tissue sections of the mice were stained with calbindin (green, Alexa Fluor 488 probed) to label the Purkinje cells in cerebellar lobes III–V. The representative images and zoomed parts of Purkinje cells are shown. Purkinje cells without fragmented neurites are indicated by arrows. The counted number of all Purkinje cells in each captured field are calculated in the bottom panel. Bar = 50 μm. Data represent mean ± s.d. of three independent experiments (total cell counts in 28 fields). * *p* < 0.05, ** *p* < 0.01, *** *p* < 0.001, **** *p* < 0.0001 (Student’s *t*-test).

The effect of *n*-BP on the alleviation of motor deficits in SCA3 was revealed, thus we addressed whether *n*-BP could improve cerebellar metabolic activity and atrophy. Previous studies reported that the SCA3 mice presented decreased metabolic activity in the central nervous system and damaged Purkinje cells as well [41,42,43]. To observe the differences between WT and SCA3, 23-week-old mice were treated for 12 weeks with *n*-BP and then subjected to in vivo positron emission tomography-magnetic resonance imaging (μPET-MRI) analysis for examining metabolic activity with 2-deoxy-2-[^18^F] fluoro-D-glucose ([^18^F]FDG) (Figure 2A). Results exhibited lower glucose metabolism, calculated as the standardized uptake value (SUV) (Figure 2A: yellow circle as ROI and quantified in Figure 2B), in the selected regions of SCA3 than WT and SCA3-BP groups (35-week-old mice). This could imply that *n*-BP significantly increased the cerebellar metabolism in the SCA3 animals, although it was not close to the WT’s metabolic activity of glucose analogue. Moreover, cerebellar size was also determined in voxels based on MRI data (Figure 2A: yellow circle as ROI and calculated in Figure 2C). Findings showed that the cerebellar atrophy was significantly ameliorated upon *n*-BP treatment that was also observed in the 28-week-old murine brain samples in a macroscopic view (Figure 2D and numbers quantified in x × y).

Next, the integrity of cerebellar Purkinje cells from mice at 28 weeks of age (5 weeks exposure to treatment) was analyzed. The tissue sections were probed with calbindin (green), used as a Purkinje cell marker [44], for the confocal microscopic analysis (Figure 2E). In the zoomed images, neurite networks in the cerebellum from four groups are shown. There were more observed Purkinje cells with non-fragmented neurites in WT and SCA3-BP animals (arrows indicated) compared to SCA3-V or SCA3 groups. Furthermore, the number of Purkinje cells in each cerebellar tissue section was quantified (Figure 2E bottom panel). These findings indicate that more Purkinje cells were protected from loss upon *n*-BP administration compared to SCA3 and SCA3-V groups. These results demonstrate that the *n*-BP prevents the Purkinje cells from loss to maintain metabolic activity and alleviate the cerebellar atrophy.

### 2.3. n-BP Promoted Autophagy to Eliminate the Aggregates In Vivo/In Vitro

**Figure 3:**
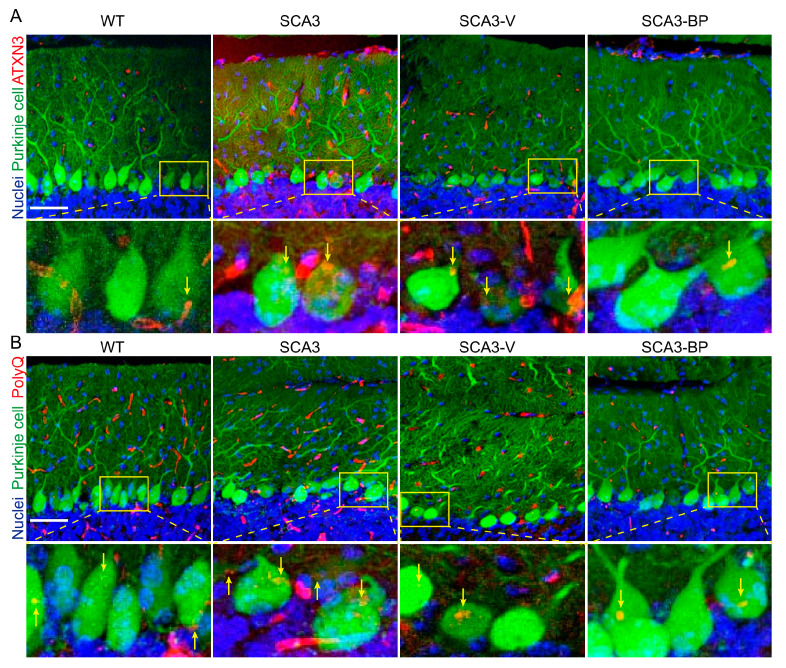

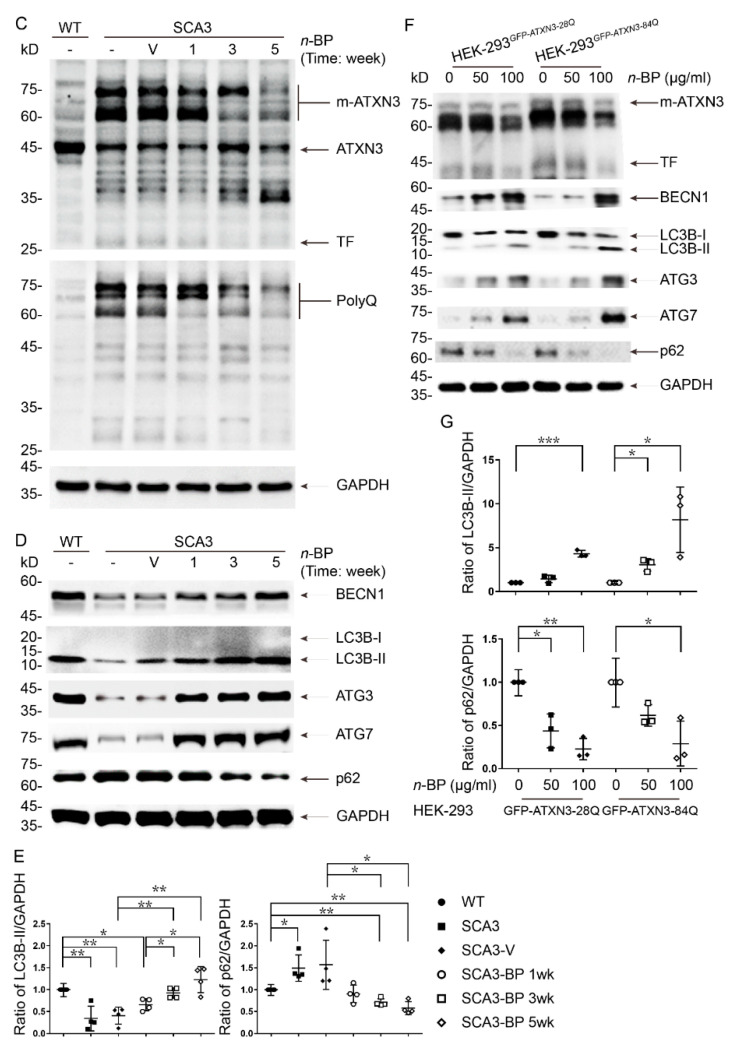
*n*-BP reduced mutant *ATXN3* aggregates and toxic fragments through promoting autophagy in vitro and in vivo. The *ATXN3* aggregates (**A**) and the polyQ proteins (**B**) (red, Alexa Fluor 594 conjugated) were located at the Purkinje cells (green, Alexa Fluor 488 labeled) in the cerebellum tissues from 28-week-old mice. Hoechst 33342 was used to label nuclei. Arrows mark the colocalization of *ATXN3*/polyQ and calbindin in the zoomed images. Bar = 50 μm. (**C**) Each sampling cerebellum was analyzed to identify the expression of mutated *ATXN3* (m-*ATXN3*) with polyQ expansion (polyQ) and toxic fragments (TFs) by Western blotting assay. *n*-BP administration could efficiently decrease the accumulation of these abnormal proteins versus vehicle or non-treated SCA3 mice. Arrow indicates the wild-type *ATXN3* at 45 kD. (**D**,**E**) The representative Western blot analysis showing the increased level of BECN1, LC3B-II, ATG3, and ATG7 and a concurrent decreased expression of p62 detected in the murine cerebellum from WT and SCA3-BP groups. Anti-LC3B, p62, and GAPDH antibodies were used on the same blot. The ratios of LC3B-II and p62 to GAPDH were calculated using ImageJ software and normalized to WT. (**F**,**G**) HEK-293 cells transfected with GFP-*ATXN3*-84Q or control GFP-*ATXN3*-28Q were treated with *n*-BP (50 or 100 μg/mL) or vehicle (0 μg/mL). After 6 h treatment, cell samples were subjected to the immunoblotting assay. Findings suggested that *n*-BP could reduce the accumulated m-*ATXN3* and TFs and the expression of p62 in HEK-293*^GFP-ATXN3-84Q^* cell models. Consistently, expression of autophagic players including BECN1, LC3B-II, ATG3, and ATG7 could be induced by *n*-BP. GAPDH, LC3B-II, and p62 were detected on the same blot. The ratios of LC3B-II and p62 to GAPDH were calculated and respectively normalized to control or HEK-293*^GFP-ATXN3-84Q^* cells. GAPDH was used as an internal control. Data represent mean ± s.d.; * *p* < 0.05, ** *p* < 0.01, *** *p* < 0.001 (Student’s *t*-test). All experiments were repeated at least three times.

The accumulation of polyQ expanded *ATXN3* or TFs can result in the loss of Purkinje cells, and these abnormal aggregates are one of the hallmarks of SCA3 [14,16,17,20]. To find out whether the *n*-BP-alleviated behavioral abnormalities were correlated with the amount of aggregates in the cerebellar tissues. Cerebellar anterior lobes from 5 weeks of drug administration in 28-week-old animals were immunostained with *ATXN3* (Figure 3A, red) or polyQ (Figure 3B, red) to mark the aggregation, and Purkinje cells and nuclei were labeled as green and blue fluorescence, respectively (Figure 3A,B). The aggregates including *ATXN3* and polyQ were observed with diffusive puncta, and some *ATXN3* and polyQ dots colocalized with calbindin (Figure 3A,B, yellow arrows). In addition, Purkinje cells containing different numbers of localized *ATXN3* or polyQ were quantified and analyzed (zoomed images and Supplemental Figure S1). Cerebellum sections from SCA3-BP or WT were found to have less colocalized *ATXN3* or polyQ with calbindin in Purkinje cells than the groups of SCA3 and SCA3-V mice. Next, three different scheduled euthanization samples of SCA3-BP (24-, 26-, and 28-weeks-old mice) were subjected to protein expression analysis and compared with samples acquired from WT, SCA3, and SCA3-V animals at 28 weeks of age (Figure 3C). The figure shows the reduction of aggregates including m-*ATXN3* and TFs in a time-dependent manner. The lowest expressions of mutant *ATXN3* and TFs were detected at the fifth week. Similar results were obtained where levels of m-*ATXN3*, including polyQ expanded form, and TFs in the cerebellum were consistent with immunofluorescent staining (Figure 3A,B and Supplemental Figure S1). Intriguingly, *n*-BP did not influence the normal *ATXN3* expression (arrow indicates 45 kD).

Treating SCA3 mice with *n*-BP helped to eliminate aggregates, and one of the protein degradation mechanisms, viz. autophagy, was highly promoted in a dose-dependent manner (Supplemental Figure S2). There are several stages during autophagic processes to form the autophagosome for the degradation of unrequired substances [29,34]. Among them, BECN1, autophagy-related protein (ATG) complex including ATG3, ATG7, and LC3B-I/II, and p62 are responsible for controlling the process in sequence. In the *n*-BP exposed SCA3 mice that were sacrificed at 24, 26, and 28 weeks of age, Western blotting analysis showed the upregulation of BECN1, ATG complex, and LC3B-II and a concurrent decreased level of p62 in a time-dependent manner (Figure 3D,E). Much more abundant autophagic players were detected in SCA3-BP and WT groups compared to animals in SCA3 and SCA3-V groups (28 weeks old). To establish an in vitro model that could mimic the responses of *n*-BP in SCA3 cerebellum, both HEK-293 cells expressing either GFP-*ATXN3*-28Q (control) or 84Q were used (Figure 3F). Reduced protein level of the GFP-*ATXN3*-84Q (m-*ATXN3* and TFs) was observed, and this could have been the consequence of autophagy promotion by *n*-BP treatment. Both cellular and murine LC3B-II or p62 to GAPDH ratios were quantified using ImageJ software (Figure 3E,G), and the findings showed that *n*-BP could enhance autophagy. The highly expressed BECN1 initiated the autophagic process, supported by the sequentially induced autophagosomal proteins LC3B-II, ATG3, and ATG7 and degraded p62 protein in either HEK-293*^GFP-ATXN3-84Q^* or HEK-293*^GFP-*ATXN3*-28Q^* cell models. These data demonstrate the notion that *n*-BP enhances autophagy by up-regulating the level of BECN1, LC3B-II, ATG3, and ATG7 in vitro and in vivo, and the abnormal aggregates are accordingly eliminated.

**Figure 4:**
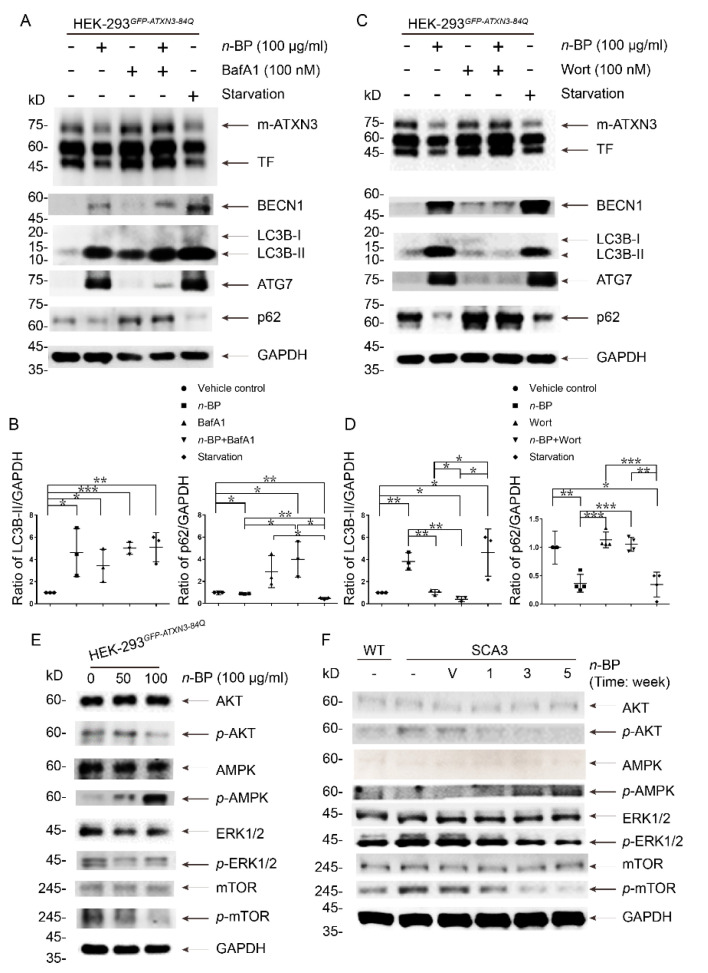
*n*-BP acted in early stage of autophagy to potentiate the elimination of aggregates through inhibiting mTOR pathway. HEK-293*^GFP-ATXN3-84Q^* cell model was used to dissect the mechanistic action of *n*-BP under Bafilomycin A1 (BafA1) (**A**) or wortmannin (Wort) (**C**) exposures. *n*-BP or starvation inducing BECN1, LC3B-II, and ATG7 expression and p62 degradation and reducing m-*ATXN3* and TF levels indicated the activation of autophagy. (**A**,**B**) Combined treatment of *n*-BP and BafA1 could not result in the reduction of m-*ATXN3* and TFs. Autophagic flux, revealed by ratios of LC3B-II and p62 to GAPDH, was measured, and the changes provided the effect of *n*-BP on autophagic promotion. (**C**,**D**) In addition, wortmannin (Wort) could inhibit the *n*-BP-induced autophagic process and retain mutant *ATXN3* proteins in the cells. The levels of LC3B-II, p62, and ATG7 were not different from vehicle control and Wort with or without *n*-BP treatment. (**B**,**D**) Ratios of LC3B-II and p62 to GAPDH were calculated and normalized to the vehicle control. GAPDH, LC3B, and p62 were detected on the same blot. (**E**) In HEK-293*^GFP-ATXN3-84Q^* cells, Western blot results showed that *n*-BP (50 or 100 μg/mL) could reduce the level of phosphorylated AKT (*p*-AKT) and ERK1/2 (*p*-ERK1/2) and induce the level of phosphorylated AMPK (*p*-AMPK), respectively. Consequently, *p*-mTOR was detected in a lower level than vehicle control (0 μg/mL). (**F**) Cerebellar tissues were sampled from WT, SCA3-BP, and vehicle or non-treated SCA3 groups (28-week-old mice) for Western blotting analysis. The expression levels of phosphorylated AKT, ERK1/2, and mTOR were lower in WT and *n*-BP treated SCA3 mice compared to SCA3 and SCA3-V groups. AMPK activation was also detected in SCA3-BP group. GAPDH was used as an internal control. Data represent mean ± s.d.; * *p* < 0.05, ** *p* < 0.01, *** *p* < 0.001 (Student’s *t*-test). All experiments were repeated at least three times.

For a more detailed molecular action of *n*-BP, the widely used Bafilomycin A_1_ (BafA1), a late stage autophagy inhibitor to block the fusion of autophagosome and lysosome [45], was used to dissect the stage of *n*-BP involvement. Using the HEK-293*^GFP-ATXN3-84Q^* cell model with *n*-BP in the presence or the absence of BafA1 (Figure 4A,B), Western blotting analysis showed m-*ATXN3* and TFs were obviously accumulated in cells co-treated with *n*-BP and BafA1. Both LC3B-II and p62 were used to evaluate autophagic flux [46], and the changes could indicate the function of *n*-BP in autophagy promotion. Administration of HEK-293*^GFP-ATXN3-84Q^* cells with BafA1 halted *n*-BP-induced autophagy; therefore, the upstream phosphatidylinositol 3-kinase (PI3K) inhibitor wortmannin (Wort) was used to reveal whether *n*-BP affected the early autophagic nucleation [47,48] (Figure 4C,D). In comparison to the vehicle control, results showed that the level of aggregated proteins was not altered post treatment with *n*-BP along with Wort. The activated autophagy by nutrient starvation leading to the amount of LC3B-II increases and p62 decreases could be observed in the immunoblotting as the positive control for the experiment. The results suggest that removal of mutants and aggregation through *n*-BP promoted autophagy are not completed under BafA1 or Wort treatment.

### 2.4. n-BP Could Be an Autophagy Enhancer through Modulating AMPK/AKT/ERK1/2 to Inhibit mTOR Pathway In Vitro/In Vivo

Our findings demonstrate that compromised autophagy in SCA3 mice can be promoted by *n*-BP to alleviate disease progression. Therefore, the mechanism of *n*-BP involvement in autophagy needs to be elucidated. First, we attempted to explore whether the removal of m-*ATXN3* and TFs in *n*-BP-induced autophagy was regulated through the mTOR pathway, one of the most important upstream signaling mediators [30,32]. HEK-293 cells harboring *GFP-*ATXN3*-84Q* with *n*-BP or vehicle treatments were analyzed (Figure 4E). Results showed *n*-BP could reduce the expression of phosphorylated AKT (*p*-AKT) and ERK1/2 (*p*-ERK1/2) and induce the activation of AMPK (*p*-AMPK). Therefore, the activated autophagy through *n*-BP treatment was the consequence of mTOR inhibition. Furthermore, Western blotting results of cerebellum from SCA3-BP animals obviously displayed levels of decreased *p*-AKT, *p*-ERK1/2, and *p*-mTOR and increased *p*-AMPK compared to SCA3-V and SCA3 groups (Figure 4F). Taken together, *n*-BP can play a role in the inhibition of mTOR via modulating AKT, AMPK, and ERK1/2 signaling pathway. Inhibiting mTOR signaling in vivo/in vitro strongly indicated the potential of *n*-BP in autophagy enhancement.

## 3. Discussion

In the discovery of *n*-BP, our team described that *n*-BP could be used before the onset of SCA3 [18] and in diseased animals displaying uncoordinated behavior and gait ataxia. Conclusive results from the previous study using 8-week-old SCA3 transgenic mice (MJD84.2) can support the potential effect of *n*-BP with the 3 month treatment on delaying SCA3 disease onset and protecting neural cells from the quinolinic acid induced neurotoxicity. Cemal et al. described that SCA3 (MJD84.2) mice from 20 weeks of age tended to lose weight and progressed with a defect in coordination and limb weakness [49]. Clinically, patients usually receive treatments or exercise program after the diagnosis of SCA3 disease. Moreover, to figure out whether a short term treatment period could show efficacy on SCA3 disease, the newly designed therapeutic regimen was tested. Therefore, the treatment protocol was changed to evaluate the efficacy on SCA3 animals with severe disease progression. The severely impaired motor functions of the SCA3 diseased animals were significantly improved by *n*-BP treatment, and they presented behaviors similar to the WT mice. Furthermore, exposure to *n*-BP increased the metabolism of glucose resulting from the prevention of loss of Purkinje cells in the SCA3 murine cerebellum. The cerebellar atrophy in SCA3 mice was accordingly mitigated. These findings attributed the obvious reduction of TFs and polyQ expanded/mutated *ATXN3* aggregation to *n*-BP that enhanced autophagy via up-regulating the protein levels of BECN1, LC3B-II, and ATG complex. To dissect the intervention and the detailed role of *n*-BP in autophagy sequences, the autophagic inhibitors, BafA1- or wortmannin-treated HEK-293 harboring **ATXN3*-84Q-GFP* cells were subjected to protein expression analysis. The results supported that the upstream signaling of mTOR, AKT, AMPK, and ERK1/2 in autophagy was modulated by *n*-BP. We proposed that the small molecule *n*-BP, functioning as mTOR inhibition, can ameliorate the progressive pathogenesis of SCA3 through the promotion of autophagy to eliminate the abnormal aggregates, thereby preventing the loss of Purkinje cells in the cerebellum.

The human *ATXN3*-84Q transgenic mice that we used in the study represented a severe progress of behavior incoordination and gait imbalance. Their disease usually began and deteriorated from 13/14-weeks-old, and neural degeneration, nerve demyelination, and axonal loss could be symptomatic and observed at 23–26 weeks of age [12,18,49]. Our data showed that *n*-BP could improve 2–3 fold in motor function of SCA3 animals at 28-week-old, although the significant difference between *n*-BP-treated SCA3 and WT mice was noted (Figure 1B). It is worth mentioning that the footprint patterns in paw overlap and stride length analysis results showed that *n*-BP treated SCA3 mice presented a similar capability of balance as in WT animals (Figure 1C,D).

Cerebellar structure of the SCA3 transgenic mice exhibited atrophy related to the loss of Purkinje cell [9,43]. In this work and in our previous publication [18], *n*-BP treatment could efficiently maintain the survival of Purkinje cell in 21- or 28-week-old animals, proven using Cresyl violet and calbindin staining of IHC/IF. However, observation of the same number of Purkinje cells in WT as compared to SCA3-BP (28-week-old) raised a question that Purkinje cells were maintained and increased. Rajamani et al. proposed the pro-proliferative capability of *n*-BP increasing calbindin expressed cells. Besides, other reports also showed that *n*-BP increased abundant motor neurons in spinal cord of SOD1^G93A^ transgenic animals (ALS mouse model) and dopaminergic neurons in α-synuclein transgenic *Caenorhabditis elegans* (Parkinson’s disease animal model) compared to vehicle control [40,50]. These two reports support the neuroprotective function of *n*-BP. Furthermore, Kang Chi et al. demonstrated that *n*-BP-treated adipose-derived stem cells (ADSCs) could enhance their expression of neural differentiation-promoting and homing factors including nuclear receptor related 1 (Nurr1), stromal-derived factor 1 (SDF1), and brain-derived neurotrophic factor (BDNF) [51]. These findings indicate *n*-BP can upregulate neurogenesis-related gene expression and may be used for application of regenerative medicine. *n*-BP administration not only performs neuroprotection through enhancing autophagy to eliminate toxic fragments but also prevents neuropathological progress of SCA3 disease. The possibility of *n*-BP playing a role in neurogenesis or neuroregeneration is not excluded.

It is generally accepted that neurodegenerative diseases, e.g., SCAs, Huntington’s disease (HD), amyotrophic lateral sclerosis (ALS), frontotemporal lobar degeneration (FTLD), and Alzheimer’s disease (AD), may be treated with the scavenger of toxic aggregates to alleviate neural cells degeneration and prevent disease progression [1,2,3,4,5,6,39,52,53,54,55]. Among them, none are approved for clinical use, but a number of therapeutic approaches are under evaluation in preclinical status [56,57,58,59] and clinical trials [60,61,62,63,64]. Many preclinical studies focused on inhibiting or dissociating the aggregation of misfolded/calpain-cleaved *ATXN3* [16,17], and patients with SCA3 generally have low expression of autophagic players [19,65]. Recently, SCA3 transgenic mice were subjected to metabolic modulation by caloric restriction followed by trehalose treatments to activate autophagy [56,57], and they provided a convenient method to delay the progression of SCA3 disease. In the metabolic control viewpoint, *n*-BP can also modulate tryptophan 2, 3-dioxygenase (TDO2) and sequentially decrease the amount of quinolinic acid and calcium influx [18,66]. Given that calpain without calcium ions is inactivated and inhibited, the Purkinje cells can be protected from the toxic effect of calpain-cleaved aggregates. The *n*-BP-mediated improvement in motor coordination performance of SCA3 mice is attributed to its dual role in regulating metabolism and enhancing autophagy.

In recent works on treating ALS disease, two groups demonstrated that daily 400–500 mg/kg *n*-BP dosing in ALS transgenic mice (SOD1^G93A^) could prolong life span, improve motor function, reduce motor neuron loss, inhibit inflammatory and oxidative stress, and suppress autophagy [39,40]. Nevertheless, *n*-BP treatment might not significantly delay ALS disease onset in the transgenic animal model. Autophagic signaling in ALS was revealed to be activated with upregulated autophagy of motor neurons within patients’ spinal cord and SOD1^G93A^ murine motor neuron [67]; however, the damaged autophagic flux was identified in the same transgenic mouse model [68]. Zhou et al. and Hsueh et al. showed decreased levels of LC3B-II, BECN-1, and p62, suggesting autophagic inhibition with phosphorylated mTOR up-regulation. On the contrary, our results showed that 50 mg/kg twice daily dosing *n*-BP in SCA3 transgenic model increased levels of BECN1, LC3B-II, ATG3, and ATG7 with a concurrent decreased p62 expression (Figure 3D,E) through inhibition of mTOR pathway (Figure 4F). The achieved autophagy process correlated with the decrease in aggregation (Figure 3A–C and Supplemental Figure S1). However, we detected some polyQ signals in WT, shown in Figure 3B,C. Based on the manual of anti-polyQ antibody, a protein containing more than 38 Q was recognized. The normal repeat contained 12–44 Q [69], and this could have been the cause of the small puncta of polyQ in Purkinje cells in WT murine cerebellum that we observed. Thus, the number of *ATXN3*/polyQ aggregates within Purkinje cells was analyzed and revealed that the cells containing aggregates more than four, either *ATXN3* or polyQ, in WT and SCA3-BP groups were less than vehicle or non-treated SCA3 mice. In brief, autophagy can be differently regulated in spinal cords of ALS and cerebellums of SCA3, and detailed therapeutic mechanism via autophagy still requires more investigations, e.g., the effect of *n*-BP on autophagic flux and aggregated proteins in ALS diseases may be analyzed.

Previously, the function of *n*-BP in decreasing levels of active calpain was addressed [18], and several reports also indicated that calpain inhibition can prevent the proteolysis of mutant *ATXN3* and lead to the reduction of fragment formation [16,17,70]. Inhibition of calpain activity could decrease the production of differently sized *ATXN3* fragments, although they used different animal models. In addition, depletion of calpastatin induced calpain activity, resulting in the accumulation of proteolytic fragments. *n*-BP treatment was used to examine whether the toxic fragment existed in HEK-293 cell lines. Data suggested protein fragments around 45 kD and 35 kD could be the signals of TFs in HEK-293*^GFP-ATXN3-84Q^* cells (Figure 3F) and SCA3 patient-derived iPSC (Supplemental Figure S3), respectively. Furthermore, SCA3 transgenic mice with *n*-BP administration showed the protein fragment with polyQ was around 27 kD (Figure 3C). We accordingly propose that *n*-BP-reduced proteins can be the TFs. Different molecular weight of these TFs is probably due to the variation between cell and animal systems.

In the period of 5 weeks of *n*-BP treatment, the band fragments around 35 kD were increased in a time-dependent manner but were not conjugated with polyQ (Figure 3C), thus it could not have been the toxic fragments. These ~35 kD protein fragments were possibly composed of *n*-terminal *ATXN3* or derived from the products of autophagic degradation. Such an N-terminus *ATXN3* without tagging polyQ stretch in the mice with genetic manipulation revealed no apparent anatomical defects at birth [71]. However, the 9-month-old homozygous N-terminal *ATXN3* mutant mice would show altered behavior and defects in motor coordination. On the contrary, a mutant *ATXN3* fragment conjugated with polyQ could lead to a SCA3-like phenotype in the mouse model [72]. While studying 35-week-old (~9-month) SCA3 transgenic mice, *n*-BP further controlled and delayed the atrophy of cerebellum (Figure 2C), and the glucose metabolic activity was even higher than in mice without treatment (Figure 2A,B). In comparison with WT, the 35 kD fragments may slightly affect the loss of cerebellar Purkinje cells associated with low glucose metabolism, which can be observed in patient with SCAs [73,74].

It remains unclear how the accumulation of abnormal aggregates impairs autophagy or whether failure to dissociate toxic aggregates is due to autophagic dysfunction. Numerous studies demonstrated that the polyQ region of *ATXN3* can interact with BECN1 to allow the conduction of autophagy [27,65]. This protein interaction enables *ATXN3* to deubiquitinate BECN1 and protects it from entering the proteasome degradation process. The SCA3 mouse model used in the report showed low expression level of BECN1 (Figure 3D), and this possibly resulted from abundant polyQ expanded *ATXN3* proteins (Figure 3C). *n*-BP treatment decreased aggregation and promoted autophagy, but wild-type *ATXN3* was not affected (Figure 3C). Moreover, several reports described that *ATXN3* can support autophagy through various mechanism. In the *atx-3* (orthologous *ATXN3*) deletion *C. elegans* model, increase of starvation-induced mortality was independent of BECN1 signaling, and the finding might be attributed to nematode orthologue *ATXN3* lacking polyQ tract [28,75]. When amino acids were not sufficient for supporting cell survival, deubiquitinase *ATXN3* was also recruited on a lysosome to interact with ubiquitinated Rheb for mTORC1 inhibition [21]. Our data may indicate that the normal function of *ATXN3* was not perturbed, and *n*-BP treatment posed no obvious harm to tested animals (Supplemental Figure S4 showed *n*-BP did not mediate the ubiquitin pathway or inhibit protein synthesis to decrease the aggregates [76]), although it was revealed that excessive autophagy may trigger cell death and contribute to neuron damage [77,78].

The detailed role of *n*-BP in autophagy was tested using HEK-293 expressing the *GFP-*ATXN3*-84Q* cell model. Treating with BafA1, an inhibitor of autophagosome maturation blocked the completion of autophagy, resulting in the accumulation of LC3B-II [45]. As shown in Figure 4A,B, protein aggregation was not removed, and TFs were further increased, because the *n*-BP-induced autophagic process was not achieved in the treatment of BafA1. The autophagic inhibitory activity of BafA1 was understood and used to measure autophagic flux, and the nutrient deprivation could be applied as a positive control in studying autophagy [46]. After a 6 h period of *n*-BP treatment or starvation (Figure 4A,C), increased levels of BECN1, LC3B-II, and ATG7 and decreased levels of m-*ATXN3*, TF, and p62 were consistent. In the presence of BafA1, all critical autophagic players were mediated and then accordingly inhibited autophagy elimination of aggregates. The changes of autophagic flux, evaluated by ratios of LC3B-II or p62 to GAPDH [79], in the *n*-BP treatment with or without BafA1 revealed that *n*-BP could be an enhancer of autophagy. Although initiation of BECN1 and ATG7 was detected under *n*-BP exposures, we speculated that such autophagic inhibition was mediated by BafA1 that blocked *n*-BP-induced autophagy and the action of *n*-BP affected the steps prior to the autophagosome formation. Therefore, wortmannin that inhibited PI3K to trap the nucleation complex in a pre-autophagosomal structure was applied [45,47]. In Figure 4C,D, results supported that wortmannin could also block the autophagic elimination induced by *n*-BP, and LC3B-II and p62 expressions were not altered as vehicle control [80].

Some drugs were developed, tested, and approved for their effect on different stages of autophagic pathway, e.g., rapamycin and metformin. Rapamycin is a specific inhibitor of mTOR that binds to mTOR and activates autophagy [31,32]. In the SCA3 transgenic mouse, one of the causes of accumulated aggregation could be the highly activated mTOR (Figure 4F). After *n*-BP administration, mTOR was found to be inhibited in both animal and cell models (Figure 4E,F). A previous study demonstrated interaction of mTORC1 and Rheb with poly-ubiquitination could contribute an amino acid induced mTORC1 activation [21]. *n*-BP eliminated polyQ expanded *ATXN3* and did not inhibit normal *ATXN3* expression, and this effect on binding of wild-type *ATXN3* and Rheb to remove ubiquitin of Rheb might further affect mTORC1 activity. In addition to mTOR inhibition, other druggable targets such as AKT, ERK1/2, and AMPK were modulated by *n*-BP (Figure 4E,F and Supplemental Figure S3). Among them, AMPK can induce autophagy by inhibiting mTOR directly or indirectly through activating upstream tuberous sclerosis 2 (TSC2) [31]. Other functions of *n*-BP were revealed, such as the activation of AMPK, i.e., phosphorylated AMPK, which inhibits vascular stenosis through mTOR inhibition [81]. Such findings of increased phosphorylated AMPK leading to down-regulation of mTOR are consistent with the results in our cell and animal models. This therapeutic strategy using AMPK activator is being evaluated in the clinical trial for treating leukemia, and the approved drug metformin may be examined [82]. In terms of the unique molecular mechanism, *n*-BP intervention may not serve as a single targeted small molecule.

We showed in SCA3 transgenic mice that administration of *n*-BP improved their motor behaviors at 5 weeks (28-week-old mice) and increased cerebellar metabolism at 12 weeks (35-week-old mice). Safety and tolerance of *n*-BP were noted by daily cage observation and had no obvious effect on body weight or general health of mice. In addition, *n*-BP treating Purkinje progenitor cells differentiated from the SCA3 patient-derived iPSCs revealed that *n*-BP could mediate AMPK/AKT/ERK1/2 signaling and inhibit mTOR to induce autophagy, resulting in reducing mutant *ATXN3* and TFs (Supplemental Figure S3). These results may imply the effect of *n*-BP on treating SCA3 patients. In the discovery and the characterization of *n*-BP, the cell cytotoxicity through inhibiting receptor tyrosine kinase, stemness, survival, and proliferation was reported [83]. Controversy may be raised in treating cancers and neurodegenerative diseases. Among the targeted therapeutic agents, AMPK activator drugs, e.g., metformin and berberine, have potential therapeutic applications in metabolic disorders, neurodegenerative diseases, and cancers [84,85,86,87,88,89,90,91]. Our recent works and this report revealed that *n*-BP can functionally activate AMPK [81], although the details of its mechanism in autophagy remain an effort to be addressed. The finding that *n*-BP can inhibit the mTOR network to promote autophagy without interfering with the wild-type *ATXN3* expression may provide a new strategy for treating polyQ diseases by regulating the molecular events associated with the elimination of toxic aggregates.

## 4. Materials and Methods

### 4.1. Animals and Treatments

SCA3 MJD84.2 transgenic female mice (C57BL/6 background) expressing the human ataxin-3 gene (*ATXN3*) with 84 glutamine repeats were used as the animal model (MGI: J:76495) to investigate the efficacy of *n*-BP [49]. The SCA3 transgenic mice were bred and provided by National Laboratory Animal Center (NLAC) (NARLabs, Taipei, Taiwan). Genotyping was confirmed using PCR (primer sequences for EXON2N forward: 5′-GAATATTTTAGCCCTGTGGAATT-3′, EXON2N reverse: 5′-GTGCGATAATCTTCACT AGTAAC-3′). Mice were housed under conventional 12 h light–dark cycles in a temperature-controlled room, and the experiments were carried out in accordance with approval and in compliance with the animal protection laws under the National Dong Hwa University Animal Management Commission for care and use of laboratory animals (NDHU-015). The animals (postnatal 23–24 weeks) received oral route administration of olive oil (vehicle) or 50 mg/kg of *n*-BP twice daily for five weeks.

### 4.2. Behavior Assays

All 23- or 24-week-old mice were pre-trained in 3–4 repeats for the rotarod performance study and the footprint test [18]. Briefly, motor coordination and balance were evaluated with a rotarod test (IITC Life Science, Woodland Hills, CA, USA). Mice were placed on the rotarod at a constant speed (training mode, 5 rpm.) for a maximum of 5 min, and then data were collected at an accelerated speed (5 to 40 rpm in 5 min). The latency to fall of each animal was recorded six times per week. To obtain the footprints per week, mice’s front- and hind-feet were painted with black, blue, green, and red colors, respectively, and the animals walked along a 100 cm long and 10 cm wide sheet of white paper for acquiring the pattern of their gait. All measurements were in centimeters. The uniformity of the step alternation was measured using the distance of left or right front/back footstep overlap. The stride length was the average distance between the center of the trail and the center of the previous trail after the sequence of four consecutive steps, excluding footprints made at the beginning and the end of the run. Each hind and front base width was evaluated as the mean distance between the left and the right trailing edges. These values were determined by measuring the vertical distance of a given step from the line connecting the front and the back steps opposite thereto. The average of five to six values per measurement was used for the analysis.

### 4.3. Animal Imaging

To evaluate cerebellar metabolic activity and size, positron emission tomography-magnetic resonance imaging (μPET-MRI) with the glucose analogue of radiotracer 2-deoxy-2-[^18^F] fluoro-D-glucose ([^18^F]FDG) was used. The used animals (postnatal 23–24 weeks) received oral route administration of olive oil (vehicle) or 50 mg/kg of *n*-BP twice daily for 12 weeks. Briefly, mice were fasted to minimize the influence of blood sugar for at least 12 h prior to intraperitoneal injection with approximately 118 μCi of [^18^F]FDG (kindly provided by National Yang-Ming University, Taipei, Taiwan). After an uptake period of 30 min, mice were scanned with a PET/MR 7T device (Bruker, Billerica, MA, USA) under 2% isoflurane anesthesia. MR scanning was performed and immediately followed by a period of 30 min acquisition of PET scanner. Intensity of T1-weighted MR images was obtained in coronal, sagittal, and transverse planes, 4-iteration and field of view (FOV): 40 mm in 256 × 256 pixels and the voxel size: 0.25 mm × 0.25 mm × 0.25 mm. All PET data were reconstructed with MR intensity images using AMIDE software (version 1.0.4) [92], and the region of interest (ROI) tool was applied. To reveal glucose analogue metabolism of cerebellum, the standard uptake value (SUV) of FDG-PET was calculated for the accumulation rate. Additionally, murine cerebellum volume was estimated in voxels.

### 4.4. Immunofluorescence (IF) Staining

Mice were euthanized in a CO_2_ chamber, and their whole brains were perfused and fixed with 4% paraformaldehyde (Sigma-Aldrich, St. Louis, MO, USA). The brain samples were cut into serial 25 μm sections using a cryostat microtome (Leica CM3050S, Wetzlar, Germany). For immunofluorescence staining, cerebellar tissue sections were blocked with 1% bovine serum albumin (BSA) blocking solution for 60 min. Permeabilized samples were probed using appropriate dilution of antibodies: anti-spinocerebellar ataxia type 3 (MAB5360, Merck Millipore, Billerica, MA, USA), anti-polyglutamine-expansion disease marker (MAB1574, Merck Millipore), and anti-calbindin (ab108404, Abcam, Cambridge, UK) in blocking solution for overnight within a humidified chamber at 4 °C. Tissue slides were then washed three times in PBS for 10 min each and incubated with fluorophore-conjugated (Alexa Fluor 594 or Alexa Fluor 488 dyes, Thermo Fisher Scientific, Waltham, MA, USA) secondary antibodies in blocking solution for 60 min at room temperature. Samples were also labeled with Hoechst 33342 to mark the nuclei, mounted with anti-photobleaching medium, and observed using confocal microscopy (Zeiss LSM510, Oberkochen, Germany). A 20× or 40× oil immersion objective was used to capture Z stack images of Purkinje cell layer (25 μm thickness), and ZEN Blue software was applied to export the images.

### 4.5. Western Blotting Analysis

Cerebellum and cell samples were lysed with RIPA solution supplemented with protease inhibitor (Merck Millipore) and phosphatase inhibitor (Merck Millipore). Proteins were separated on sodium dodecyl sulfate-polyacrylamide gels (SDS-PAGEs) and then transferred onto nitrocellulose (NC) membranes (Merck Millipore). After blocking with 5% BSA in tris-buffered saline containing 0.1% Tween 20 (TBS-T) at room temperature for 1 h, membranes were then incubated with anti-spinocerebellar ataxia type 3 (MAB5360, Merck Millipore), anti-polyglutamine-expansion disease marker (MAB1574, Merck Millipore), anti-Akt (9272, Cell Signaling, Beverly, MA, USA), anti-phospho-AKT-(Ser473) (9271, Cell Signaling), anti-AMPKα (2532, Cell Signaling), anti-phospho-AMPKα-(Thr172) (2535, Cell Signaling), anti-phospho-p44/42 MAPK (Erk1/2, Thr202/Tyr204) (4370, Cell Signaling), anti-mTOR (ab32028, Abcam), anti-mTOR (phospho S2448) (ab109268, Abcam), anti-beclin 1 (pab12473, Abnova, Taipei, Taiwan), anti-ATG3 (ab108251, Abcam), anti-ATG7 (ab133528, Abcam), anti-LC3B (ab51520, Abcam), anti-SQSTM1/p62 (ab109012, Abcam), anti-ubiquitin antibody (ab134953, Abcam), or anti-GAPDH antibody (GTX100118, GeneTex, Hsinchu, Taiwan) at 4 °C for overnight. Membranes were washed 3 times for 10 min each with TBS-T, followed by incubation with the corresponding horseradish peroxidase (HRP)-linked secondary antibody: anti-mouse (ap192p, Merck Millipore) or anti-rabbit (ap187p, Merck Millipore) for 1 h at room temperature. Western blotting signals were visualized using enhanced chemiluminescent substrate (Merck Millipore) and imaged with LAS-3000 (Fujifilm, Tokyo, Japan).

### 4.6. Cell Culture and Treatment

Human embryonic kidney (HEK)-293*^GFP-ATXN3-28Q^* and HEK-293*^GFP-ATXN3-84Q^* were established in house [18]. Briefly, the endogenous expression of ataxin-3 gene (*ATXN3*) in HEK-293 cells was low, thus pEGFP-C1-*ATXN3*Q84 or -Q28 were transfected into HEK-293 for establishing an in vitro SCA3 model. The cells were cultured in Dulbecco’s modified Eagle’s medium (DMEM) with 1% L-glutamine (200 mM stock solution) (HyClone, GE Healthcare Life Science, South Logan, UT, USA), 10% fetal bovine serum (FBS) (HyClone), and 1% penicillin/streptomycin (HyClone) and incubated in 5% CO_2_ at 37 °C. Cells were plated in culture dishes to a confluency of 80% before treatments with *n*-BP (10, 50, or 100 μg/mL, Everfront Biotech Inc., Taipei, Taiwan), MG132 (50 μM, 474790, Sigma-Aldrich), cycloheximide (CHX) (100 μg/mL, 239763, Sigma-Aldrich), wortmannin (100 nM, ab120148, Abcam), or Bafilomycin A_1_ (100 nM, ab120497, Abcam) dissolved in dimethyl sulfoxide (DMSO). Nutrient deprivation was conducted using DMEM without 10% FBS to culture the cells. Starvation or drug treatments were conducted for 6 hours.

### 4.7. Quantitative Real-Time PCR

Total RNA was extracted using RNeasy Mini Kit (Qiagen, Valencia, CA, USA), and reverse transcription was performed using a SuperScript III First-Strand Synthesis System (Thermo Fisher Scientific) according to the manufacturer’s instructions. Gene expression was quantified by real-time quantitative PCR using a FastStart Essential DNA Green Master (Roche, Basel, Switzerland). The primer sequences were as follows: BECN1: forward 5′-GGCTGAGAGACTGGATCAGG-3′, reverse 5′-CTGCGTCTGGGCATAACG-3′; ATG3: forward 5′-GTCCCTTATTAGAGAAACTGTAT-3′, reverse 5′-AGAATATGACTACACAAGACACT-3′; ATG7: forward 5′-GTGACTGTGCTTTCAGGT-3′, reverse: 5′-GGATGTGACTGCTTGTTC-3′; LC3B: forward 5′-ACCATGCCGTCGGAGAAG-3′, reverse 5′-ATCGTTCTATTATCACCGGGATTTT-3′. All reactions were performed in triplicate.

### 4.8. Statistical Analysis

All data were statistically analyzed with Graph Pad Prism (v.5.0). *p* < 0.05 is considered to be statistically significant (Student’s *t*-test).

## Supplementary Materials

The following are available online at https://www.mdpi.com/article/10.3390/ijms22126339/s1.

## Abbreviations

AKT	Protein kinase B
AMPK	AMP-dependent protein kinase
ATG	Autophagy-related protein
BafA1	Bafilomycin A_1_
ERK	Extracellular signal-regulated protein kinase
HEK-293	Human embryonic kidney-293
LC3B	Light-chain-3 B subunit
MJD	Machado-Joseph disease
mTOR	Mammalian target of rapamycin
MRI	Magnetic resonance imaging
PET	Positron emission tomography
SCA	Spinocerebellar ataxia
WT	Wild type
