# Supplementary material for: n-Butylidenephthalide Modulates Autophagy to Ameliorate Neuropathological Progress of Spinocerebellar Ataxia Type 3 through mTOR Pathway
Source: Int J Mol Sci. 2021 Jun 13;22(12):6339. doi: 10.3390/ijms22126339 (PMC8231882; doi:10.3390/ijms22126339)
Supplement: Supplementary file 1 [file ijms-22-06339-s001.zip › ijms-1231379-supplementary.pdf]

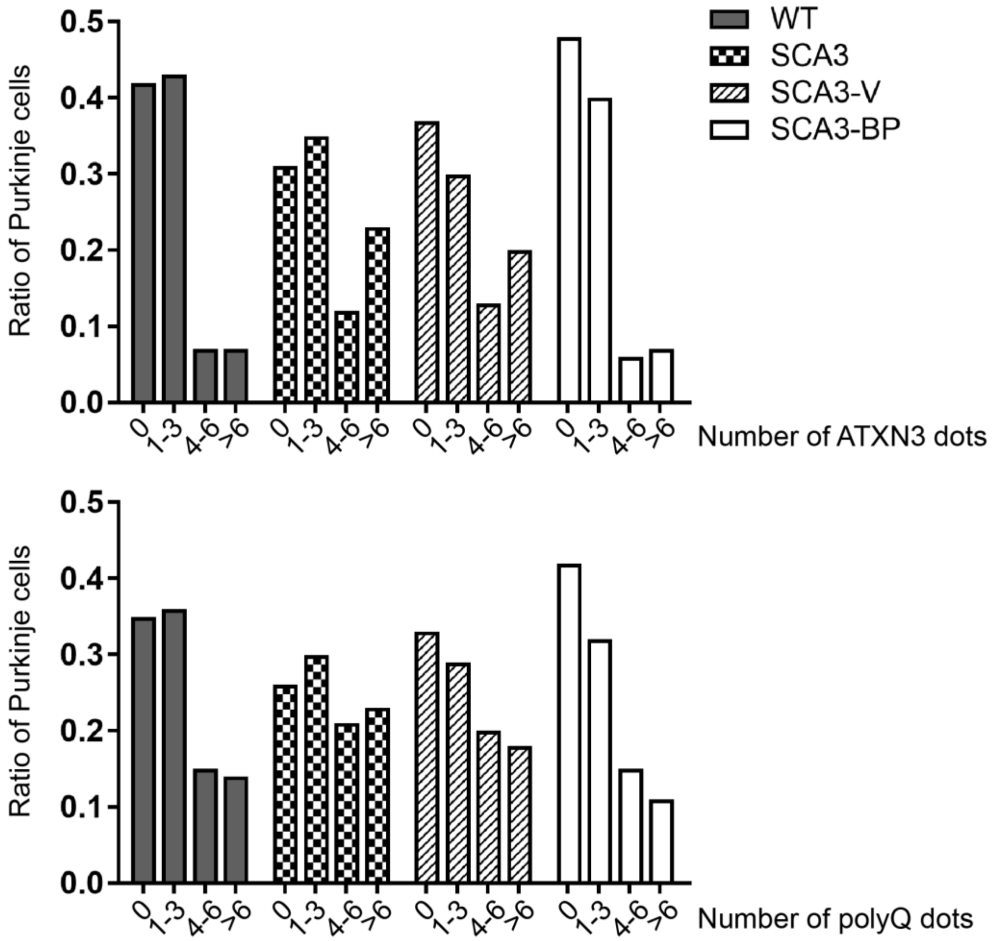

Supplemental Figure S1.

Quantification of ATXN3 or polyQ protein aggregates in calbindin expressed Purkinje cells.

Cells having both green (calbindin) and red (ATXN3: *n* = 449 and polyQ: *n* = 403 total counted cells) fluorescent dyes (Figure 3A and B), indicative of Purkinje cells with aggregates, were identified by confocal microscopy (orange dots) and counted as ratio of Purkinje cells in each group. Purkinje cells contained different numbers of the protein aggregates, so data were further classified into four populations to reveal the trend. Findings suggested that Purkinje cells containing more than 4 colocalized dots of ATXN3 or polyQ with calbindin were observed more abundant in the SCA3 and SCA3-V groups, compared with the WT and *n*-BP treated SCA3 mice. The histogram indicates

that *n*-BP treatment can effectively inhibit the accumulation of protein aggregates in SCA3 transgenic mice.

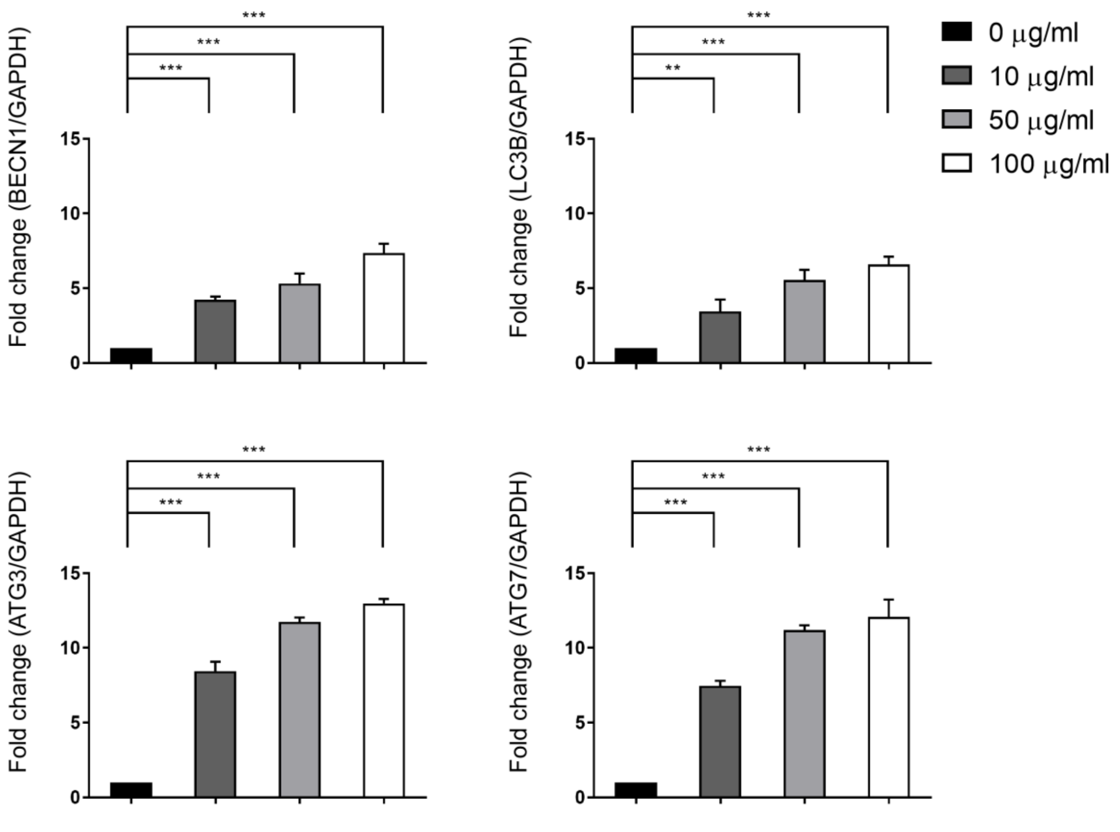

Supplemental Figure S2.

Gene expression of autophagic markers in *n*-BP treated HEK-293$^{GFP\text{-}ATXN3\text{-}84Q}$ cells.

Q-PCR analyzed these HEK-293 cells harboring GFP-ATXN3-84Q with indicated *n*-BP treatment. Findings revealed that *BECN1*, *LC3B, ATG3* and *ATG7* gene expression were significantly increased after 24-hr treatment with *n*-BP. GAPDH was used as an internal control for fold change. N = 3 independent experiments (each 3 technical replicates). Data represent mean ± s.d.; * $p < 0.05$, ** $p < 0.01$, *** $p < 0.001$ (Student's *t*-test).

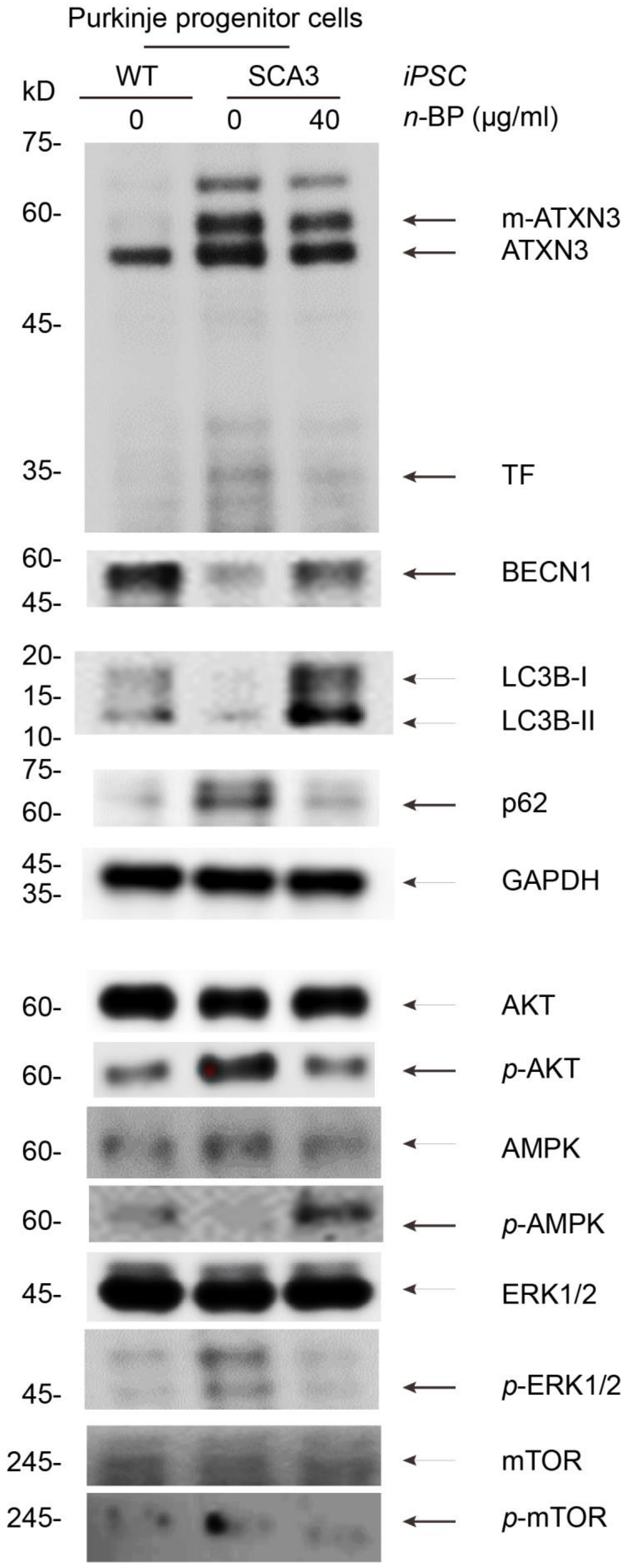
Purkinje progenitor cells
WT
SCA3
iPSC
kD
0
0
40
n-BP (μg/ml)
75-
60-
45-
35-
m-ATXN3
ATXN3
TF
60-
45-
BECN1
20-
15-
10-
LC3B-I
LC3B-II
75-
60-
p62
45-
35-
GAPDH
60-
AKT
60-
p-AKT
60-
AMPK
60-
p-AMPK
45-
ERK1/2
45-
p-ERK1/2
245-
mTOR
245-
p-mTOR

Supplemental Figure S3.

***n*-BP modulated AMPK/AKT/ERK1/2 signaling to inhibit mTOR and potentiated autophagy for the elimination of mutant ATXN3 and toxic fragments in SCA3 patient-derived iPSCs.**

Western blotting assays analysis of Purkinje progenitor cells, differentiated from iPSCs of the healthy individual (WT) or SCA3 patient (https://catalog.bcrc.firdi.org.tw/, materials were acquired from Bioresource Collection and Research Center (BCRC), Food Industry Research and Development Institute, Hsinchu, Taiwan) with or without indicated *n*-BP 24-hr treatment. In comparison with vehicle treated SCA3 Purkinje progenitor cells, *n*-BP could reduce aggregates including m-ATXN3 and TFs. Such findings implied that *n*-BP promoted autophagy through inducing the expression of BECN1 and LC3B-II and degradation of p62. Moreover, decreased level of phosphorylated AKT (*p*-AKT) and ERK1/2 (*p*-ERK1/2) and activation of AMPK (*p*-AMPK) were triggered by *n*-BP. It suggested that *n*-BP could accordingly inhibit mTOR (*p*-mTOR) pathway to induce autophagy. GAPDH was used as an internal control. N = 2 experiments.

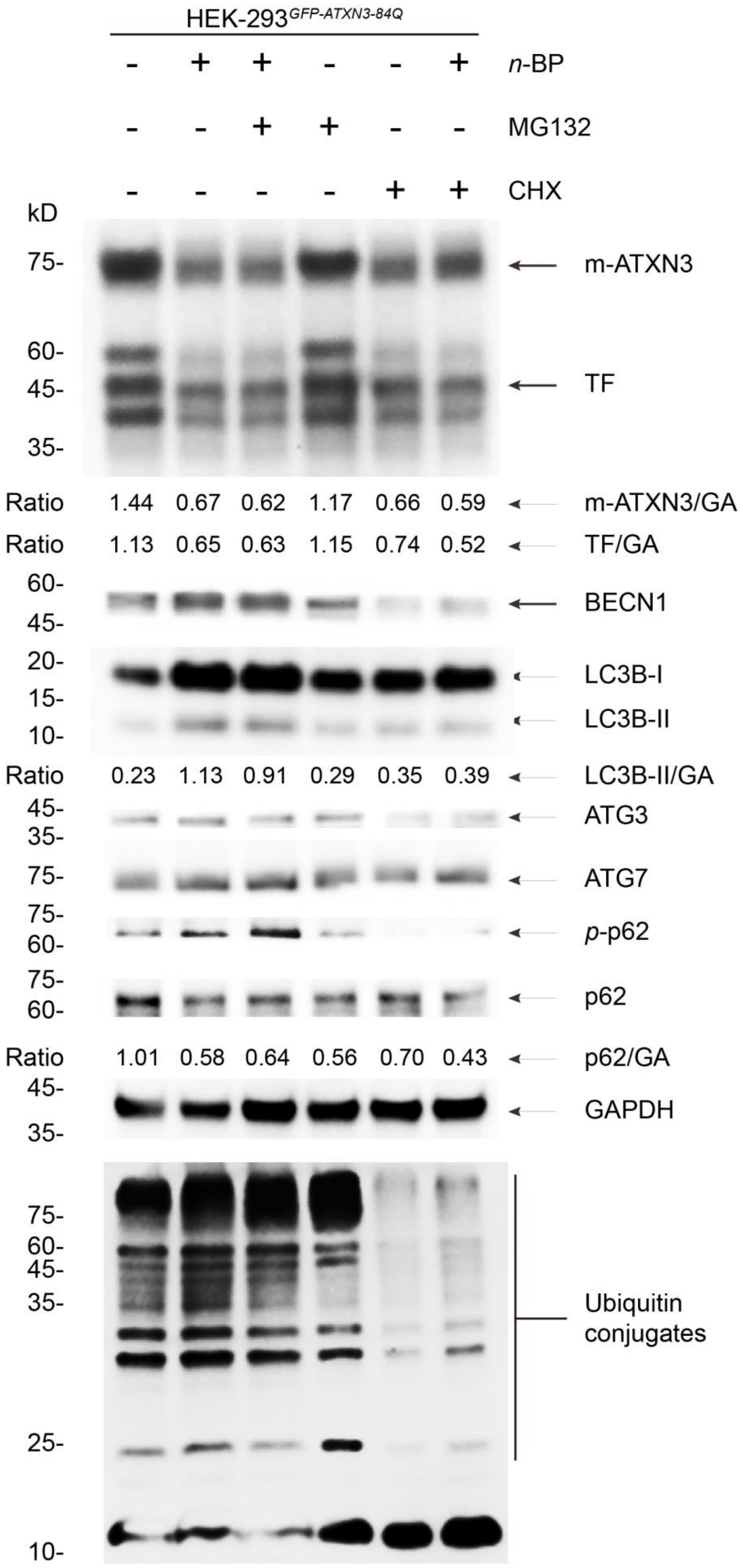
HEK-293 GFP-ATXN3-84Q
- + + - - + n-BP
- - + + - - MG132
- - - - + + CHX
kD
75-
60-
45-
35-
m-ATXN3
TF
Ratio 1.44 0.67 0.62 1.17 0.66 0.59 m-ATXN3/GA
Ratio 1.13 0.65 0.63 1.15 0.74 0.52 TF/GA
60-
45-
BECN1
20-
15-
10-
LC3B-I
LC3B-II
Ratio 0.23 1.13 0.91 0.29 0.35 0.39 LC3B-II/GA
45-
35-
ATG3
75-
ATG7
75-
60-
p-p62
75-
60-
p62
Ratio 1.01 0.58 0.64 0.56 0.70 0.43 p62/GA
45-
35-
GAPDH
75-
60-
45-
35-
25-
10-
Ubiquitin conjugates

Supplemental Figure S4.

***n*-BP-induced elimination of aggregates was not achieved by modulating proteasome degradation pathway or inhibiting protein synthesis.**

Western blotting assays analysis of HEK-293$^{GFP\text{-}ATXN3\text{-}84Q}$ cells with or without indicated *n*-BP, MG132 (proteasome inhibitor) or cycloheximide (CHX, protein synthesis inhibitor) treatments. *n*-BP treatment along with or without MG132 induced expression of BECN1, LC3B-II, ATG3, ATG7 and *p*-p62 and reduced level of p62, compared to control and MG132 alone groups. This promotion of autophagy via *n*-BP that enhanced the elimination of aggregates was independent of proteasome degradation pathway. Intriguingly, increased BECN1 protein level could be detected under MG132 treatment, compared to control. Additionally, higher total levels of ubiquitin conjugates in response to MG132 were revealed, in comparison with control. After administration of CHX in the presence or absence of *n*-BP, decreased level of BECN1, LC3B-II, ATG3, ATG7 and *p*-p62 were detected, compared to *n*-BP alone group. It suggested that reduction of polyQ expanded ATXN3 (m-ATXN3) and TFs may be a result of the inhibitory effect of CHX on protein synthesis. Besides, CHX could block starvation-induced autophagy but not mTOR inhibitor-induced autophagy [76]. In combining *n*-BP and CHX treatments, data showing that levels of m-ATXN3 and TFs could be slightly lower than CHX or *n*-BP only groups. Ratios of m-ATXN3, TF, LC3B-II and p62 to GAPDH was analyzed using ImageJ software. GAPDH, LC3B and p62 were detected on the same blot. GAPDH was used as an internal control. N = 2 experiments.
